# Right Ventricular Perforation and Cardiac Tamponade Post-FlowTriever® System Treatment in High-Risk Pulmonary Embolism: A Rare Complication

**DOI:** 10.7759/cureus.81796

**Published:** 2025-04-06

**Authors:** David Casasola-González, Leyre Alonso-Gonzalo, Tatiana Pire-García, Pablo Demelo-Rodríguez, Sergio Moragón-Ledesma, Rubén Alonso-Beato, Jorge García-Carreño, María Eugenia Vázquez-Álvarez, Lucía Ordieres-Ortega, Francisco Galeano-Valle

**Affiliations:** 1 Venous Thromboembolism Unit, Department of Internal Medicine, Hospital General Universitario Gregorio Marañón, Madrid, ESP; 2 School of Medicine, Universidad Complutense de Madrid, Madrid, ESP; 3 Sanitary Research Institute Gregorio Marañón (IiSGM), Hospital General Universitario Gregorio Marañón, Madrid, ESP; 4 Department of Cardiology, Hospital General Universitario Gregorio Marañón, Madrid, ESP

**Keywords:** catheter-directed thrombectomy, flowtriever® system, percutaneous mechanical thrombectomy, pulmonary embolism, right ventricular perforation

## Abstract

Catheter-directed thrombectomy (CDT) using the FlowTriever® system offers promising results and could represent a paradigm shift in reducing morbidity and mortality of patients with intermediate-high and high-risk acute pulmonary embolism (PE). However, it is crucial to be aware of the risks and limitations associated with this technique.

We present an extremely rare case of a 44-year-old male patient diagnosed with cerebral venous thrombosis of the transverse and sigmoid venous sinuses complicated by hemorrhagic lesions, who developed a high-risk PE within 24 hours of admission. Due to concomitant contraindications for anticoagulation in a patient with intracranial hemorrhage, the FlowTriever® system was chosen as the preferred intervention despite its associated risks. CDT was performed, provoking right ventricular perforation and cardiac tamponade immediately after the procedure.

Only one previous case has been reported describing this extremely rare complication following FlowTriever® CDT. We reviewed the literature on indications, efficacy, and safety of this procedure, as well as our experience with this technique in patients with intermediate-high- and high-risk PE, clarifying whether prior reports included only FlowTriever® or other CDT devices. Additionally, we discuss the clinical implications of this case, highlighting the importance of careful patient selection when considering CDT in high-risk PE cases with contraindications for anticoagulation.

## Introduction

The initial management of patients with acute pulmonary embolism (PE) hinges on short-term mortality risk stratification (within the first 30 days), which relies on hemodynamic status, the presence of right ventricular (RV) dysfunction, and assessment using the Pulmonary Embolism Severity Index (PESI) scale [1].

Systemic fibrinolysis is the first-line treatment in patients diagnosed with acute PE presenting with hemodynamic instability (high-risk PE) [1]. Due to the lack of high-quality evidence, primary reperfusion using catheter-directed thrombectomy (CDT) is not currently the first-line treatment for patients with high-risk acute PE. Instead, according to current guidelines, CDT should be considered for patients with high-risk PE in whom fibrinolysis is contraindicated or has failed [2]. CDT should also be considered a rescue treatment for initially stable patients in whom anticoagulant treatment fails, i.e., those who experience hemodynamic deterioration despite adequate-dose initial anticoagulation [2].

CDT techniques, including catheter-directed clot fragmentation, mechanical embolectomy, local thrombolysis, and combined pharmaco-mechanical approaches, act primarily by relieving the thromboembolic obstruction of the proximal pulmonary arteries, restoring pulmonary blood flow and improving RV function [2].

The main goal of aspiration embolectomy is to remove embolic material and prevent further embolization. Thrombus aspiration is achieved by applying suction (manually or using a dedicated system) through large bore catheters.

The FlowTriever® system (Inari Medical, Irvine, CA) is an aspiration system designed for interventional treatment of PE. The access point for the FlowTriever® system is the femoral vein or internal jugular vein. This system differs from other CDT devices by providing a filtration mechanism that allows for thrombus removal while returning blood to the patient [2,3], thereby potentially reducing the risk of complications associated with other methods. FlowTriever® system rapidly improves RV dysfunction, despite having a potentially lower chance of bleeding complications compared to fibrinolytic therapy. However, it has yet to be systematically studied against other catheter-based therapies or against systemic fibrinolysis [3].

To date, three studies have evaluated the safety and efficacy of the FlowTriever® system in patients with acute PE. These are the FLAME study (a prospective, multicenter, nonrandomized, parallel-group observational study of high-risk PE), the FLARE study (a single-arm prospective study in patients with intermediate-high-risk PE), and the FLASH (a multicenter, prospective registry enrolling up to 1,000 US and European PE patients with intermediate-high- and high-risk acute PE).

While most common adverse effects reported in these studies were related to hemoglobin decrease and did not include reports of cardiac injuries or pulmonary vascular injuries [4-6], complications such as RV perforation, although extremely rare, represent a novel and serious concern not typically associated with the use of the FlowTriever® system or similar devices.

It is worth mentioning that two randomized controlled trials, the STORM-PE trial and STRIKE-PE study, are currently ongoing, comparing catheter-directed therapies with anticoagulation alone in patients with intermediate-high-risk acute PE [7,8].

Subsequently, we present a case of a patient with high-risk PE treated with the FlowTriever® system who suffered an RV perforation, an exceptional complication with this procedure. Patient consent was obtained.

## Case presentation

**Figure 1:**
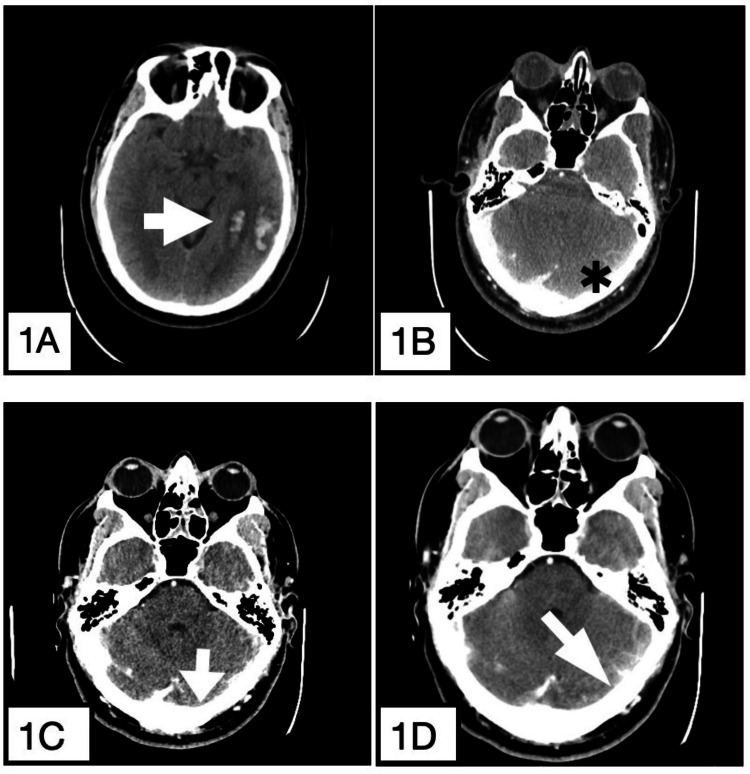
(A) A cranial computed tomography (CT) scan showing two hemorrhagic lesions in the left temporal-occipital territory without underlying vascular lesions (white arrow). (B) Transverse and sigmoid venous sinuses and the left Labbé vein asymmetry (black asterisk). (C) A venous phase extension confirmed that parenchymal hemorrhages were caused by venous infarction after thrombosis of the transverse and sigmoid venous sinuses and the left Labbé vein (white arrow) with (D) the absence of enhancement of those sinuses previously mentioned on the left side. The vein of Labbé is also not identified on the left side.

A 44-year-old male with a history of arterial hypertension and a prior episode of acute PE in April 2020, provoked by COVID-19-infection, was not currently receiving anticoagulant treatment. He was brought to the Emergency Department in November 2022 after a tonic-clonic seizure at home, preceded by a five-day history of altered behavior. On arrival, his Glasgow Coma Scale score was 7, with reactive isocoric pupils and no focal neurological deficits. A cranial computed tomography (CT) scan showed two hemorrhagic lesions in the left temporal-occipital region, with no underlying vascular abnormalities, along with asymmetry of the transverse and sigmoid venous sinuses and the left Labbé vein. A venous-phase CT confirmed that the parenchymal hemorrhages were caused by cerebral venous thrombosis (CVT) due to thrombosis of the transverse and sigmoid venous sinuses and the left Labbé vein (Figure 1). Sigmoid sinus thrombosis occurs in 30-40% of CVT cases [9], Labbé vein thrombosis in 15% [10], and non-hemorrhagic infarcts in 20%. Due to his altered mental status, the patient was admitted to the intensive care unit (ICU) for airway management and neurological support.

**Figure 2:**
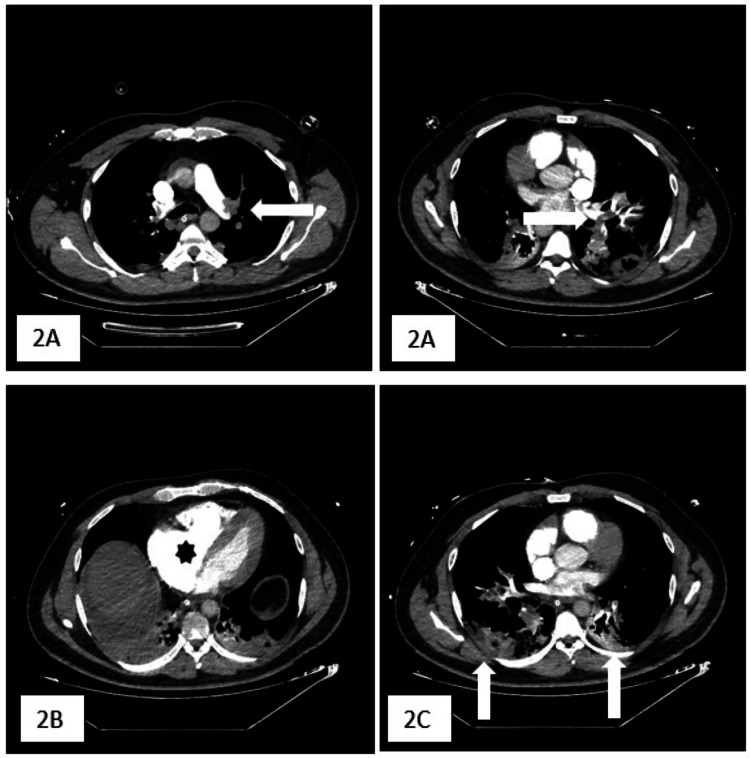
(A) Pulmonary artery computed tomography angiography confirmed extensive bilateral pulmonary embolism (horizontal white arrows) with (B) signs of right ventricular strain (black asterisk) and (C) pulmonary infarctions in the lower lobes (vertical white arrows).

Within 24 hours of ICU admission, the patient experienced hemodynamic deterioration requiring vasopressor support. Bedside transthoracic echocardiography (TTE) revealed a moderately dilated RV with systolic dysfunction and septal flattening. Laboratory tests showed a platelet count of 90,000/µL, total bilirubin of 2.9 mg/dL, creatinine of 4.8 mg/dL, high-sensitivity troponin I of 7,000 ng/mL (normal laboratory range [NR] in males: <34 ng/mL), Nt-proBNP of 1,450 ng/mL (NR <600 ng/mL), and D-dimer of 15,000 ng/mL (NR <500 ng/mL). The markedly elevated D-dimer level made it unreliable for ruling out PE. High-sensitivity troponin I and Nt-proBNP levels were elevated, indicating pressure overload and/or RV dysfunction. Pulmonary artery CT angiography confirmed extensive bilateral PE with a significantly reduced RV/left ventricular (LV) ratio (<0.9) and pulmonary infarctions in the lower lobes (Figure 2). A Doppler ultrasound of the lower extremities ruled out deep vein thrombosis.

Given the hemodynamic instability secondary to refractory obstructive shock and severe RV failure, along with multiorgan failure and contraindication for fibrinolysis due to intracranial hemorrhage, the patient was connected to an extracorporeal membrane oxygenation (ECMO) device. Concurrently, anticoagulation with unfractionated heparin (UFH) was initiated, and CDT was performed using the FlowTriever® System with ultrasound-guided puncture of the left femoral vein. A Swan-Ganz balloon catheter was used, as recommended, to prevent damage to the subvalvular apparatus and avoid perforation.

During thromboaspiration of the left main pulmonary artery, the device became unstable while switching to the right artery, migrating toward the atrium and requiring repositioning. A multipurpose catheter was then used, but it prolapsed into the RV, leading to RV perforation due to guidewire misplacement during advancement. The procedure time was 139 minutes.

Immediately afterward, bedside echocardiography revealed pericardial effusion with cardiac tamponade, requiring emergent pericardiocentesis. Surgical exploration confirmed active bleeding and perforation of the RV apex, necessitating RV repair and direct extraction of extensive clots from both pulmonary arteries. Thrombi were removed from the right main pulmonary artery, which had not been extracted percutaneously, and residual thrombotic material from the left pulmonary artery. Thrombectomy of the lobar branches was then performed using a Fogarty catheter.

In the immediate postoperative period, the patient exhibited severe RV dysfunction, requiring ongoing ECMO support, vasopressors, and continuous renal replacement therapy. No cardiac arrest occurred. ECMO was discontinued after eight days. One month later, RV function improved, but follow-up TTE revealed persistent moderate RV dilation for six months.

After four months in the ICU, he was stabilized, weaned off supportive measures, and transitioned from UFH to weight-adjusted low molecular weight heparin (LMWH), allowing transfer to a conventional ward. Upon transfer to Internal Medicine, the patient showed favorable progress with resolution of pericardial effusion and ventricular dysfunction. Neurologic symptoms resolved without focal deficits, leading to hospital discharge on apixaban 5 mg b.i.d. He has remained asymptomatic after one year of follow-up.

## Discussion

High-risk PE is a serious condition associated with poor short-term prognosis, necessitating rapid reduction of RV afterload in its management. Current treatments available for this purpose include systemic fibrinolysis, catheter-directed reperfusion techniques, and surgical thrombectomy. Additionally, ECMO is helpful in high-risk PE to maintain the circulation and oxygenation of vital organs during acute RV failure and cardiogenic shock. Hemodynamic stabilization with mechanical support offers the potential to provide the additional time necessary to better plan further management [1,2,6].

Systemic fibrinolysis has demonstrated a reduction in mortality rates in patients with high-risk PE compared to standard anticoagulation, being considered the first-line treatment [1]. However, the use of systemic fibrinolysis is restricted by the bleeding risk, and cases of therapeutic failure have been described. These recommendations stem from studies such as PEITHO trial, where systemic fibrinolysis was associated with a lower risk of hemodynamical instability but at the expense of increased risk of severe extra- and intracranial hemorrhage [11]. Given these limitations, there is an opportunity to improve outcomes, with percutaneous treatments options offering new mechanisms for clot reduction with a possible improved safety profile compared with systemic fibrinolysis [12].

The best treatment strategy for intermediate-high-risk acute PE remains unclear and controversial because of the difficulty in predicting which patients will deteriorate, although the majority will remain stable and can be treated safely with anticoagulation [1].

According to current guidelines, catheter-directed reperfusion techniques should be considered for patients with high-risk PE in whom fibrinolysis is contraindicated or has failed or as rescue treatment for initially stable patients (i.e., intermediate-risk PE) in whom anticoagulant treatment fails [2,13].

In recent years, catheter-directed techniques, with or without the use of local fibrinolytic agents, have gained importance. Since the first publications on the use of mechanical thrombectomy in the 1990s, there has been a development of multiple CDT devices. The FlowTriever® aspiration system is one of the most widely used, along with the Indigo® Thrombectomy (Penumbra, Inc., Alameda, CA) and Angiovac/AlphaVac® (Angiodynamics, Inc., Latham, NY) [11]. However, studies on CDT are small-scale, observational with heterogeneous inclusion criteria and include patients not meeting the clinical guidelines recommendations (hemodynamically stable acute PE) [14-16].

Several prospective studies have shown promising safety and efficacy results for the FlowTriever® System, although they lack a comparative arm [17]. For instance, the FLASH registry demonstrated a favorable safety profile and hemodynamic improvement after evaluating its use in 800 patients with intermediate-high (76.7%) and high-risk (7.9%) acute PE or contraindication to fibrinolysis (32.1%), with no device-related deaths and all-cause mortality rates of 0.3% and 0.8% at 48 hours and 30 days, respectively [6].

Two prospective, multicentric, single-arm studies have been fulfilled to evaluate the efficacy and safety of CDT in patients with intermediate-high-risk PE, including a total of 225 patients (the FLARE study assessed the FlowTriever® system and the EXTRACT-PE trial evaluated the Indigo aspiration system). In both, the primary endpoint was the reduction of RV/LV index measured by thoracic CT 48 hours after the procedure. Both showed a significant reduction of this parameter with CDT. However, long-term results were not evaluated, and CDT was not compared with standard treatment with anticoagulants. Both studies showed lower major bleeding rates with CDT than systemic fibrinolysis (1.7% for the FlowTriever® system and 0.94% for the Indigo® Aspiration system). Some of the reported adverse effects were respiratory failure, injury of pulmonary vessels, and myocardial injury. However, the reported procedure-related RV perforation was zero [4,18].

The FLAME study was a prospective, multicenter, non-randomized, parallel group, observational study of high-risk PE that included 53 patients treated with FlowTriever® CDT (FlowTriever® arm) and 61 patients treated with contemporary therapies (Context arm). Context arm patients were primarily treated with systemic thrombolysis (68.9%) or anticoagulation alone (23.0%). In-hospital mortality occurred in 1.9% patients in the FlowTriever arm and in 29.5% patients in the Context arm. Among the serious adverse events related to the treatment device, 1/53 patient suffered obstructive shock and 1/53 patient had RV failure. Again, the reported procedure-related RV perforation was zero [5].

Moreover, the ongoing STORM-PE and STRIKE-PE trials are pivotal in further evaluating the effectiveness of catheter-directed therapies compared to anticoagulation alone in intermediate-high-risk PE [7,8]. The inclusion of data from the PEERLESS trial, which compared CDT and mechanical thrombectomy in intermediate-high-risk PE, will also contribute to this knowledge base [19].

A limitation of the FlowTriever® system is the size and stiffness of the catheter, which may be challenging to progress in distal pulmonary arteries. Additionally, there is a learning curve necessitating experienced operators for optimal outcomes [15]. When comparing the complication profile of the FlowTriever® system with other CDT systems, such as Indigo® and Angiovac/AlphaVac®, it is important to note that these systems may also carry risks [15].

RV perforation is an infrequent but serious complication of CDT, including procedures using the FlowTriever® system. It can occur during the manipulation of the catheter or guidewire, particularly in cases where the catheter is stiffer or when excessive force is applied. Possible mechanisms include catheter stiffness, guidewire manipulation, or RV dilation and wall thinning due to the elevated pressures associated with PE. Distal pulmonary artery engagement may also increase the risk of perforation as operators navigate through challenging anatomical structures. Based on the experiences outlined in this case, several technical precautions can be suggested to minimize the risk of RV perforation. These include meticulous guidewire manipulation, avoiding forceful catheter advancement, and ensuring optimal patient positioning. The importance of the learning curve for the safe use of the FlowTriever® system cannot be overstated [16]. Therefore, it is advisable that such procedures be performed in high-volume centers with experienced operators.

Management of RV perforation involves a multi-step approach, including prompt recognition of the complication, decision-making regarding emergent pericardiocentesis, and potential surgical repair if indicated. Early intervention can significantly affect patient outcomes in such critical situations. Long-term follow-up is essential not only to assess RV function recovery but also to ensure ongoing anticoagulation and surveillance for potential late complications such as pericardial adhesions.

Thus far, only one case of RV perforation following FlowTriever® CDT has been reported in a 45-year-old woman with intermediate-high-risk PE who, similar to our case, developed cardiac tamponade post-procedure [20]. This highlights the need for greater awareness and caution in practice and underscores the importance of accumulating case reports to help inform safety guidelines and training protocols for FlowTriever® use in high-risk PE patients.

## Conclusions

In conclusion, CDT using the FlowTriever® system offers promising results and could represent a paradigm shift in reducing morbidity and mortality of patients with intermediate-high- and high-risk acute PE. However, without robust evidence from large-scale studies, establishing its safety and efficacy remains challenging. It is crucial to recognize the risks and limitations of this technique, including RV perforation - a rare but serious complication that should be suspected in cases of post-procedural pericardial effusion. Operator expertise, along with early detection and prompt management, is essential to improving patient outcomes. Accumulating case reports like ours could inform future updates to procedural guidelines or device-specific safety protocols.
